# Primary health care case management through the lens of complexity: an exploratory study of naturopathic practice using complexity science principles

**DOI:** 10.1186/s12906-022-03585-2

**Published:** 2022-04-15

**Authors:** Kim D. Graham, Amie Steel, Jon Wardle

**Affiliations:** 1 Australian Research Centre in Complementary and Integrative Medicine, Faculty of Health, University of Technology, Sydney. 15 Broadway St, Sydney, NSW Australia; 2 National Centre for Naturopathic Medicine, Southern Cross University, Military Road, Lismore, NSW 2480 Australia

**Keywords:** Primary health care, Complexity science, Naturopathy, Network mapping and analysis

## Abstract

**Background:**

Advances in systems science creates an opportunity to bring a complexity perspective to health care practices and research. While medical knowledge has greatly progressed using a reductionist and mechanistic philosophy, this approach may be limited in its capacity to manage chronic and complex illness. With its holistic foundation, naturopathy is a primary health profession with a purported alignment with a complexity perspective. As such this pilot study aimed to investigate the application of complexity science principles, strategies, and tools to primary health care using naturopathy as a case study.

**Methods:**

A network mapping and analysis of the naturopathic case management process was conducted. Mind maps were created by naturopathic practitioners to reflect their clinical conceptualisation of a common paper clinical case. These mind maps were inputed into *Gephi*, a network mapping, exploration, and analysis software. Various layouts of the data were produced, and these were analysed using exploratory data analysis and computational network analysis.

**Results:**

Seven naturopathic practitioners participated in the study. In the combined network mapping, 133 unique elements and 399 links were identified. Obesity, the presenting issue in the case, was centrally located. Along with obesity, other keystone elements included: systemic inflammation, dysbiosis, diet, the liver, and mood. Each element was connected on average to 3.05 other elements, with a degree variation between one and 36. Six communities within the dataset were identified, comprising: the nervous system and mood, gastroinstetinal and dietary factors, systemic inflammation and obesity, the endocrine system and metabolism.

**Conclusions:**

This pilot study demonstrates that it is feasible to apply a complexity science perspective to investigating primary health care case management. This supports a shift to viewing the human organism as a complex adaptive system within primary health care settings, with implications for health care practices that are more cognisant with the treatment of chronic and complex conditions and research opportunities to capture the complex clinical reasoning processes of practitioners.

**Supplementary Information:**

The online version contains supplementary material available at 10.1186/s12906-022-03585-2.

## Background

In recent decades the scientific advances of systems science [1, 2] has opened a pathway for evolving primary health care practices and research beyond a reductionist framework to incorporate a complexity perspective. Systems science is an interdisciplinary field of enquiry into the complex systems that exist in nature and other domains. Biomedicine to date has flourished using a paradigm of mechanism and reductionism to simplify the complex interactions and systems-based functioning of the human organism to reduced parts [3] deemed to operate according to linear relationships [4, 5]. This approach has enabled significant development of knowledge and treatment options for managing health [2]. However, with increasing levels of chronic and complex illness contributing to the global burden of disease [6], the limitations of a reductionistic approach for engaging with the complete, integrated and complex human system is becoming recognised [7–9].

**Fig. 1:**
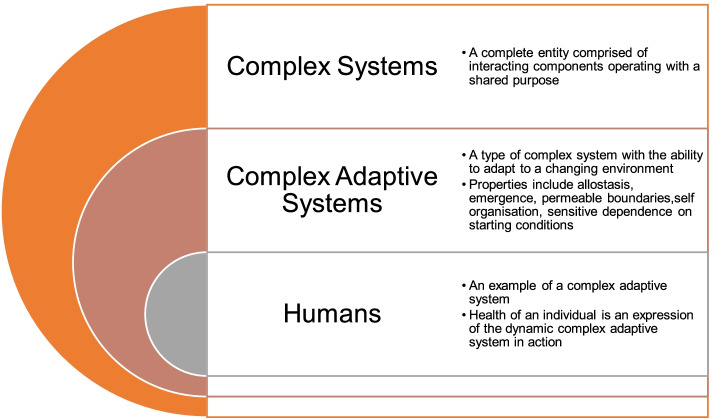
The relationship between complex systems, complex adaptive systems, and the human organism

The study of complex systems generally arises from a desire to explore and understand real systems [10], and is the basis of this exploratory descriptive pilot study. The human organism is a complex adaptive system (CAS) (Fig. 1), which is both composed of and functioning within numerous self regulating and interacting systems, including cellular, biochemical, physiological, psychological [11], as well as political, socioeconomic, and environmental [12]. Complexity science offers an alternative to the linear and mechanistic perspective that is pervasive in health care management [11], enabling a contemporary holistic insight into the health and possible treatment of the patient. The complex and integrative context and physiology of living organisms creates diagnostic and management challenges when there is over reliance on a reductionist paradigm [9, 13]. Complexity science has been posited as a way to move beyond siloistic and specialism approaches to multi-morbidity leading to a deeper understanding of practice and its possibilities [14]. In this study, we have presented a case mapping and analysis as an exemplar for how a complexity science perspective may be utilised to explore the case management process.

### Medical philosophy

All systems of medicine are based on a philosophical paradigm. While biomedicine is founded on reductionism and mechanism [15], traditional systems of medicine such as naturopathy are founded on holism [16, 17]. This philosophical position influences the understanding of the nature of being (ontology), the means used to gather knowledge, and the distinction made between acceptable beliefs and opinions (epistemology), as well as how medicine ought to be practiced (norms) [18]. However, philosophical positions are not fixed and have the potential to evolve [19]. A long held world view of ultimate connection between all elements in nature existed traditionally (for example, [20]) – in essence a form of systems thinking – until a shift occurred in the 1600 s towards a paradigm of reductionism and mechanism [3] which over time came to dominate scientific thinking. Complexity science, the study of complex systems, has been emerging over the past 100 years and this trend has accelerated over the past two decades [21] with increasing interdisciplinary focus [22]. The widespread incorporation of systems thinking into scientific endeavours challenges the position of reductionism as the primary paradigm for conducting scientific research, particularly when investigating a complex system such as the health of the human organism.

### The essence of complexity

The essence of complexity can be summed up by the statement: *the whole is greater than the sum of its parts* [21, 23]. Complexity scientists view the world as ultimately connected, and within this vast web of connections various systems can be defined. Systems thinking is a mindset where systems are considered as whole dynamic and relational entities, and not just as a composite of parts. A system is whole within itself and comprised of components that are interacting for a shared purpose [1]; systems are embedded in other systems [24] and their permeable boundaries [8] allow for exchange and interaction between them. A reductionist paradigm is potentially inadequate to comprehensively explore, understand and engage with complex systems and their inherent interactions [9, 13] and, given that the human organism is a complex system, this may inhibit improvements in the quality and safety of medical treatment and disease management.

### Human health as a complex adaptive system

CAS, a common form of complex system, have the capacity to learn and adapt in the face of changing environments [25]. While linear systems occur rarely [13], CAS are ubiquitous in nature [22]. The human organism is an example of a CAS, defined by its capacity to respond functionally to environmental change [26] and to produce novel emerging structures and properties as a result [27]. Within this paradigm, the health of the human organism may be viewed as an adaptive response to the environment in which it is immersed. CAS are whole self perpetuating systems that nest within multiple systems [24]. They are dynamic, constantly evolving, and exhibit unpredictable emergence, driving an internal process of self organisation [24]. As well as each human organism functioning as a CAS, human health also exhibits these same properties and it is the unified, whole and complex human system operating in a state of allostasis that forms the basis of health [28]. Human organisms respond to disease and treatment in a complex way [13]. While engaging with human health in a reductionistic, linear and mechanistic manner has proved a valuable strategy for advancing medicine, the adoption of complexity science and systems thinking potentially offers opportunities to further explore and better support human health [1]. The aim of this study is to investigate how complexity science principles and strategies may lead to insights capable of informing clinical reasoning and healthcare research.

### Naturopathy: a case study in complexity

Naturopathy is a traditional system of medicine practiced in over 40 countries [29] with its roots stretching back thousands of years [30]. It is recognised by the World Health Organisation (WHO) as “the general practice of natural health therapies” via naturopathic practitioners’ integration of traditional knowledge with current understanding of health and the human system [31]. Naturopathy is a *whole medicine system*; a complete medical system of theory and practice that has evolved alongside or independently from biomedicine [24]. Naturopathic philosophies (*holism* and *vitalism*) and principles (*treat the cause, treat the whole person, doctor as teacher, above all do no harm, promote health and wellbeing, prevent disease,* and *the healing power of nature*) form the basis of how naturopathy is currently taught and practiced [30, 32].

**Fig. 2:**
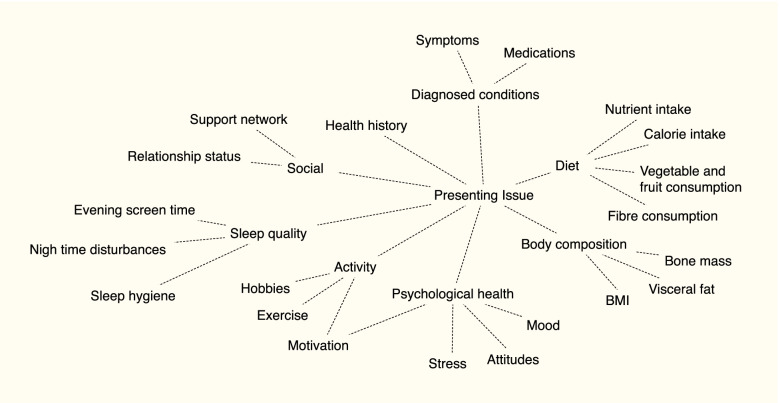
Mind map example

Naturopathy is philosophically consistent with complex, dynamic and extended network models [23, 24] based on its meta-theoretical precepts such as holism and non-specificity that it shares with other complementary medicine professions [17]. Naturopathy is founded on an inter-systems approach [33, 34] aligning it with complexity principles This philosophical foundation makes naturopathy an ideal model to test systems-based tools and strategies in a clinical healthcare setting. The holistic perspective of naturopathy may be observed in the mind maps created by some naturopaths as part of their case management process. Mind map training is a feature of naturopathic education in a number of Australian teaching institutions, and a number of naturopaths continue using mind maps into their professional clinical practice. Mind mapping is a knowledge management tool—by using a mind map, knowledge can be organised, assessed, translated and transferred—offering a means for conceptualising the presenting state of a dynamic system, and could also be considered a basic form of network mapping [35]. A mind map enables interacting elements and the relationships between the elements deemed relevant to the case to be identified [35]. An example of the type of information potentially contained in a mind map is presented in Fig. 2. The aim of this study is to use systems congruent tools and strategies (mind maps, exploratory data analysis, network mapping, and network analysis) to explore the viability of employing a complexity approach to investigate clinical reasoning in primary healthcare provision.

## Methods

### Study design

This study was conducted as a network mapping and analysis of the naturopathic clinical case mangagement process, following the method published in Graham et al. [35], and an overview of this method is outlined here. The aim of this exploratory study is to investigate the application of complexity science principles, strategies, and tools to the case conceptualisation process as an initial foray into the possibilities that a systems mindset may offer health care. In order to trial a complexity approach to understanding diagnostic clinical decision making and case conceptualisation, a paper case study (supplementary file 1) was presented to seven Australian naturopathic practitioners at different career stages who each created a case schematic in the form of a mind map. These mind maps were then amalgamated using *Gephi*, an open source network mapping, exploration and analysis software [36]. *Gephi* was used in this study to manage, visualise and analyse the data contained in the mind maps. Various layouts were implemented in order to graphically alter the presentation of the data. The network mappings created in *Gephi* underwent an exploratory data analysis (EDA) process, before being analysed using the mathematical and computational analysis tools of *Gephi*.

### Ethics approval

Ethics approval was obtained from the University of Technology, Sydney Human Research Ethics Review Committee (approval number: ETH20-4864).

### Participants, setting, and data collection

Participants responded to a social media recruitment campaign conducted via professional naturopathic Facebook groups. All participants were required to hold Bachelor-level naturopathic qualifications, currently be in naturopathic clinical practice, and be a member of a professional association that accredits naturopaths in Australia. All participants were required to routinely make use of mind maps to conceptualise their patient cases as part of the health management process. Interested participants were provided with an information sheet and required to sign an informed consent form. Each participant was emailed the same paper case study, and were requested to independently create a mind map, based solely on the information contained in the case study and using their preferred process and style, and software generated or drawn by hand, depending on their preference. While the case study was fictional, it contained information which was consistent with the type of information gathered during a standard naturopathic consultation [33]. Once created, the seven mind maps were emailed back to the research team. The data contained in these mind maps was inputed into *Gephi* and various layouts were created from this dataset.

### Data visualisation

Three types of network mappings were created using *Gephi*: a *force based attribute layout*, a *modularity layout*, and a *condensed modularity layout*. These are outlined in greater detail below. Within the *Gephi*-generated network mappings there were two aspects depicted: edges (links) and nodes (elements). The elements were represented by circles, and the links by lines. Each element included in the network mappings was an aspect of the case study identified by one or more of the participants as being relevant to their case conceptualisation process. The links signified connection between the elements, and were representative of any form of influence or relationship between the elements as identified by the participants. In the network mappings, the links were directional; the curve of each link travelling clockwise denoting the direction of influence. The size of each element indicated the number of connections it had: the larger the element, the more connections incoming or outgoing.

#### Force based attribute layout

The first layout algorithm was force based in that linked nodes attracted each other, and non-linked nodes repelled, resulting in the most connected elements clustering in the centre and those least connected being pushed to the extremities. In the force based layout, the elements identified by the participants were coloured according to five different attribute types. The attribute types were assigned by the research team and were: presenting issue, environmental influence, sign / symptom, hypothetical risk, and organ or functional sub-system (Table 1).

**Table 1:** Attribute element key

Element Colour	Attribute	Example(s)
Pink	Presenting issue	Obesity
Green	Environmental influence	Diet, alcohol intake, excessive sweating, flat mood, poor nutrient profile
Purple	Sign or symptom	Headaches, anxiety
Blue	Hypothetical risk	Thyroid function, low zinc, high cholesterol
Orange	Organ or functional sub-system	Liver, nervous system, hypothalamic–pituitary–adrenal axis, gastrointestinal system

#### Modularity and condensed modularity layouts

The second layout created was based on modularity. In this graphical representation, a *Gephi* algorithm was implemented which decomposed the network mapping into cluster based communities. Community clustering was based on the linkage patterns within the network – densely linked elements formed cliques, while less densely elements were separated. Elements were no longer coloured according to attribute, but instead by the colour of the community of which they were a member of. The third layout created was a condensed version of the modularity mapping and was comprised only of the elements with seven or more connections, in order to generate a simpler image on which to conduct exploratory data analysis.

### Data analysis

#### Exploratory data analysis

An exploratory data analysis (EDA) approach was taken with the three different network mappings of the dataset (*force based*, *modularity* and *condensed modularity*) in order to generate novel information based on the relationships amongst the data and the structure of the network mapping. EDA is a method of looking at visual representations of a dataset in order to gain insights [37], and provides an opportunity to explore a data set without preconceptions in order to glean information about the phenomena under investigation [38]. EDA is a process of exploration and “graphical detective work” [37]. In this study, this exploratory process was not intended to be confirmatory, but rather to generate novel insights about the dataset.

#### Network analysis

The network mappings were then analysed using various algorithms available within *Gephi*. This analysis was conducted at node level, such as degree, distance, and betweenness centrality, as well as at network level, such as network diameter, average degree, average path length, average clustering co-efficient and modularity. By mathematically analysing the links between various elements, it is possible to define the shortest path between any two elements (distance), the shortest path between the furthest two elements in the network (diameter), the frequency an element appears on the shortest path between two elements (betweenness centrality), the level of interconnectedness within a network (average clustering co-efficient), and the capacity of the network to decompose into communities or subgroups (modularity). Table 2 defines key network terms and measures relevant to this study.

**Table 2:** Key term and measure definitions in relation to this network

Term	Definition	Information this provides about the node or network	Significance in this network	Example or value in this network
*Foundational*				
Node	An element of a network	Identifies different components within a system	Identifies a pertinent aspect of a case presentation as determined by one or more participants	Obesity
Link	A association (direction specific) between any pair of nodes	Identifies relationships of influence within a system	Identifies a connection between two nodes that is considered relevant to the case presentation determined by one or more of participants	Alcohol intake to Liver
Path	A series of links connecting any pair or group of nodes	Identifies a sequence of connections between two or more elements	Identifies a sequence of relationships between two or more nodes as determined by one or more participants	Increased cortisol to sympathetic nervous system activation to social anxiety to excessive sweating
Cluster or Community	A substructure or clique of nodes that are more closely connected to each other compared with nodes outside the subgroup	Identifies well connected cliques within the network, revealing underlying network structure	Establishes the node subgroups as identified by the participants	Green cluster (nervous system, flat mood / depression, fatigue, low motivation, antidepressant medication, headaches, low serotonin)
*Node level measures*				
Degree	The number of links (in or out) that a node has	Identifies nodes deemed most connected with other nodes	Establishes the nodes the practitioners deemed most interactive within the network	High degree: diet, Low degree: loose stools
Average degree	The average number of links across all nodes	Establishes the average number of links across the nodes	Provides a mid-point from which the number of links each node has can be compared against	Average = 3.05 (with variation between one and 36)
Distance	The number of links on the shortest path between two nodes	Detects the minimum number of steps influence needs to travel	Establishes the intermediate steps for influence to spread between two nodes, as identified by the participants	Indoor job to compromised vitamin D status to flat mood / depression to OR alcohol intake to liver to reduced fat metabolism to oxidative stress
Betweenness centrality	How often a node appears on the shortest path between other pairs of nodes	Measures the number of paths that pass through a particular node	Demonstrates the value of each nodes in terms of its potential to interact with others as determined by the participants	Dysbiosis is on the shortest path between: diet & bloating with cramping; gas production & gut fermentation; toxin recycling & halitsosis Flat mood = 2880 Obesity = 2348 Dysbiosis = 2080 Systemic inflammation = 1648 Liver = 1248
Clustering co-efficient	The number of links a node has divided by the total number of possible links. The highest possible value is 1 (where a node is linked to all other node)	Along with the mean shortest path, the clustering co-efficient can indicate a ‘small-world’ effect, and signifies how embedded nodes are within their neighbourhood	Denotes the extent to which nodes are linked within the network	Average clustering co-efficient = 0.114
Eigenvector centrality	Measures the value of each nodes, based on the number of links it has, and the number of links the nodes it connected to has, and so on across the network	A measure of the influence of an element within the network	Denotes the extent to which well-linked nodes are connected to other well-linked nodes	Liver (0.39), hypertension (0.41), systemic inflammation (0.48), dysbiosis (0.60), obesity (0.68), and flat mood / depression (1)
*Network level measures*				
Diameter	The shortest pathway between the two most distant nodes	Provides a measure of the parameters of the network	A measure of how tightly the nodes in the network are linked, as identified by the participants	Diameter = 9
Average path length	The average of the shortest path between all pairs of nodes	The average minimum number of links between all pairs of nodes	Indication of the ease with which change can move across the system	Average path length = 3.71
Average clustering co-efficient	The average of the clustering co-efficient for all nodes	Averaged across all nodes, the proportion of nodes with a direct link to each node, divided by the total number of nodes identified in the network	A measure of how connected the network is	Average clustering co-efficient = 0.114
Modularity	A measure of the extent the network decomposes into subgroups or cliques	Reveals underlying layers of structure within the network	Within communities denser interactional possibilities exist, plus reveals potential sub-structures as identified by the participants	6 communities detected with between 12 to 33 elements each Modularity score = 0.40

## Results

Seven Australian based naturopathic practitioners participated in the study. Years of experience ranged from two to 11 years, with an average of 5.43 years.

### Exploratory data analysis

#### Force based attribute mapping

**Fig. 3:**
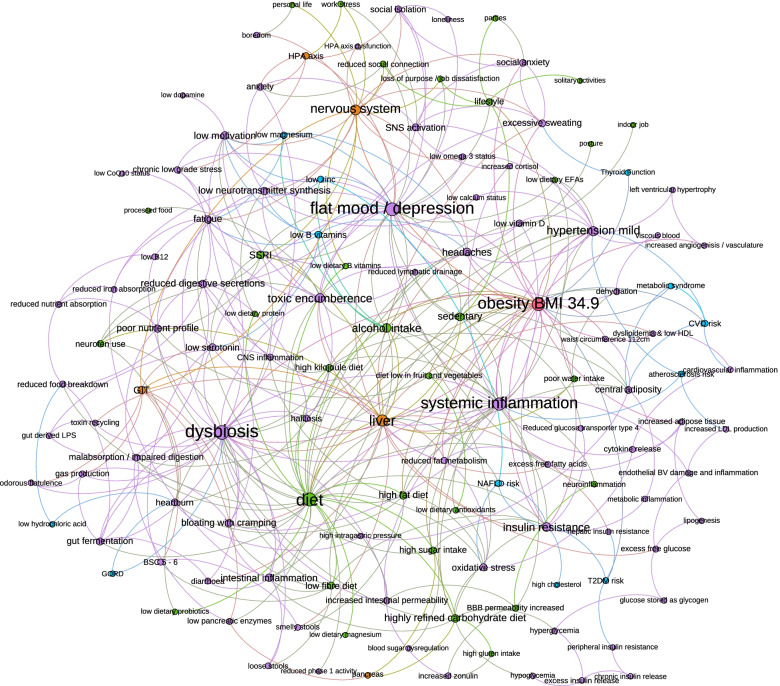
Force-based attribte mapping

The merged network mapping of the seven individual mind maps, coloured according to attribute and using a force-based layout, contained a total of 133 unique elements and 399 links (Fig. 3). Obesity, the presenting issue in the case study, was central to the mapping indicating that it was well connected to other elements within the mapping as determined by the participants. The elements the participants identified as the most integral to this case study, as indicated by size of the element and central position, were: obesity, systemic inflammation, dysbiosis, diet, the liver, and the influence of mood on these various factors.

#### Modularity mapping

**Fig. 4:**
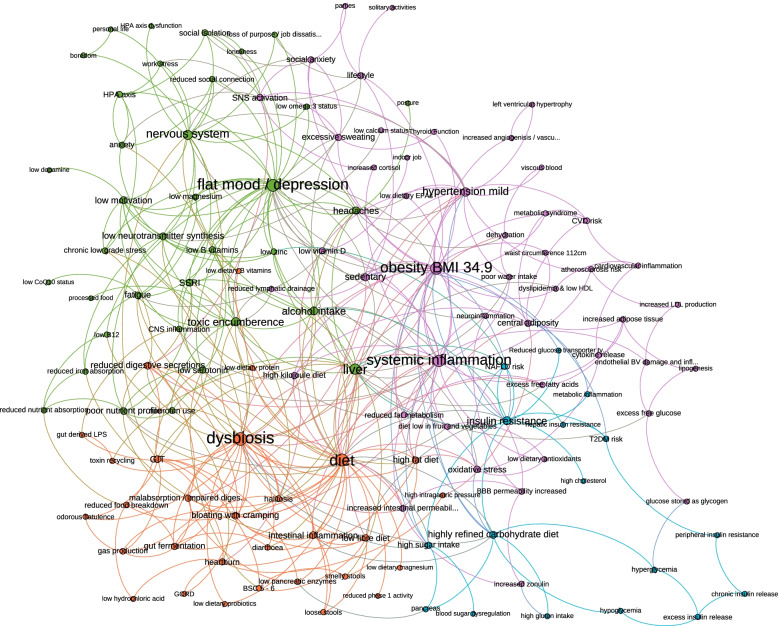
Modularity mapping

An alternative layout based on modularity represented the community structure within the network as identified by a *Gephi* algorithm, where the more densely connected nodes were clustered and colour coordinated (Fig. 4). Modularity indicates the capacity of the network to decompose into subgroups or communities. Six communities were identified by the *Gephi* algorithm, demonstrating the cliques of subsystems, organs, symptoms and environmental influences that the practitioners deemed to be most closely in relationship. The primary clusters from these six were: green (predominantly nervous system and mood elements), orange (predominantly gastrointestinal and dietary factors), pink (predominantly relating to systemic inflammation and obesity), and blue (predominantly endocrine and metabolic aspects), revealing underlying layers of structure within the network.

#### Condensed modularity mapping

**Fig. 5:**
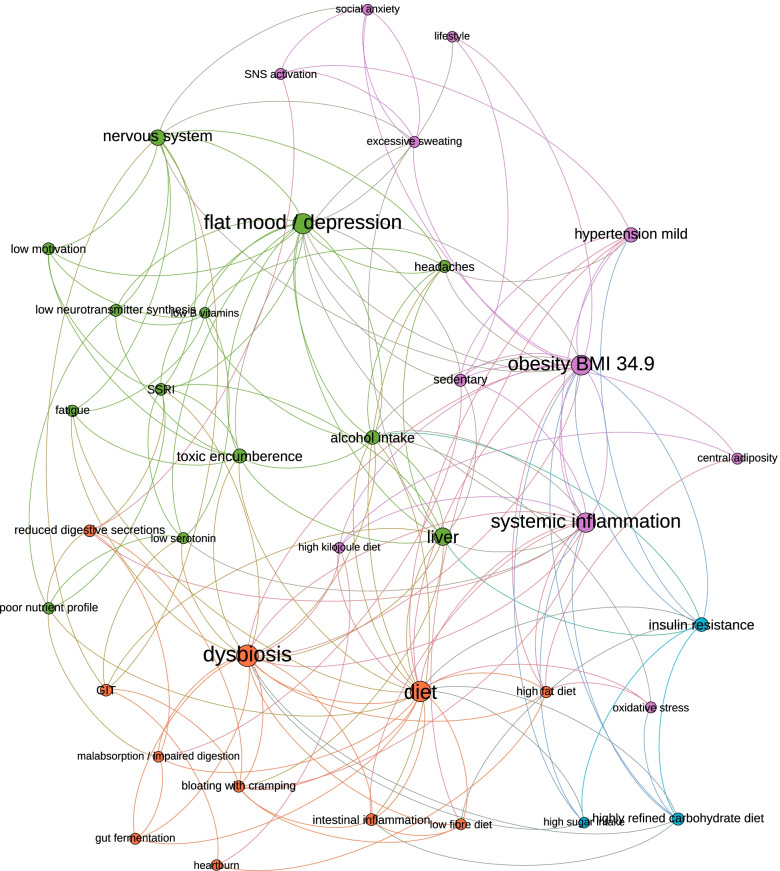
Condensed modularity mapping

A condensed modularity mapping where all elements with six links or less were removed provided information on the most highly connected elements identified by the participants, and ‘cleaned’ the image (Fig. 5). Thus, the more important elements of the mapping were preserved becoming more apparent. In this instance, the communities coloured green (predominantly nervous system and mood elements), orange (predominantly gastrointestinal and dietary factors), and pink (predominantly relating to systemic inflammation and obesity) were the primary communities retained indicating that these were the sub-structures deemed by the practitioners to be of primary relevance to the ‘patient’ presented in the paper case study.

### Network analysis

#### Network analysis: node level measures

Each element was connected to an average of 3.05 other elements, with variation in degree between one and 36, and a degree distribution pattern that was skewed to the left (supplementary file 2). Betweenness centrality, the frequency an element appears on the shortest path between any other pair of elements is shown on a distribution plot with a table of the elements with the highest values (supplementary file 3), and indicates the extent that an element might act as a bridge or intermediary between elements. The majority of elements appeared up to 500 times on the shortest pathway between any other pair of elements. The liver and systemic inflammation appeared between 1,000 and 2,000 times and obesity (the presenting issue), flat mood / depression and dysbiosis appeared more than 2,000 times each.

Eigenvector centrality is a measure of the importance of each element, based on the number of links the element has and the number of links their connections have, measured across the network. It is a measure of ‘popularity’ indicating importance based on who their connections are linked to, and not just the immediate elements that an element is linked to. A distribution of eigenvector centrality appears in supplementary file 4. The highest eigenvector value elements in the mapping included the liver (0.39), hypertension (0.41), systemic inflammation (0.48), dysbiosis (0.60), obesity (0.68), and flat mood / depression (1).

#### Network analysis: network level measures

The diameter of the network was nine, indicating the parameters of the network. The average path length (the average minimum distance between any two elements) was 3.71; phrased differently there are on average 3.71 degrees of separation between the elements. The average clustering co-efficient with a possible range of zero to one is a measure of the density of the network, and in this network meausured 0.114. Therefore, in this network each element was linked on average to 11.4% of other elements in the network.

Using the *Gephi* modularity algorithm, six communities in total were detected in the network. These can also be seen visually in the modularity mapping. Within a community, there is increased interactional potential with possible structural aspects of the network revealed. The size distribution of the communities ranged from 12 to 33 elements. The modularity score of this network was high at 0.40, indicating a decidedly connected internal structure with a high density of internal connections inside communities, measured against the links between communities, as compared with a low modularity score which would indicate clusters that were more disparate.

## Discussion

This study demonstrates that it is viable to graph clinicians’ reasoning of a primary healthcare case in the form of a network mapping, and to identify and examine its structural characteristics using exploratory data analysis and network analysis tools. In doing so, clinical reasoning is made overt enabling it to be examined by both researchers and clinicians; this novel depiction potentially enables new knowledge and insights. The application of a complexity science perspective to clinical management also supports paradigmatic integration, facilitating the assimilation of reductionist and holistic philosophies. This may expediate a shift in clinician thinking from linear, mechanistic and reductionist to one cognisant of the human organism as a complex adaptive system (CAS) while retaining the knowledge gains afforded by a reductionist and mechanistic approach. Software such as *Gephi* allows this process to be scaled up so that in addition to exploring a single case presentation as demonstrated here, multiple cases could be simultaneously explored to more thoroughly investigate a condition or pattern of symptoms, or even to create a more comprehensive mapping of the human organism. According to Newman [39], the first step in studying a network is to create a representation of it, the second step is to conduct various forms of analysis on it, and the third step is to create a model of the processes that occur in the networked system. In this paper preliminary work has been conducted on steps one and two, offering the first practical application of complexity tools to examine the clinical reasoning process of health professionals.

### Implications for practice

A complexity science perspective potentially enables innovative insights to emerge regarding the primary causality of the condition, perpetuating factors, potential future risks, and prospective individualised treatment targets. In this study, particular elements were identified as being keystones due to the centrality of their position in the force based mapping, the importance of their connections, the value of their mediary positions, and their size relative to other elements. These keystone elements are not just integral to the case presentation, they are potential leverage points within the CAS on which to focus treatment efforts in order to initiate the most positive outcomes. Additionally, the directional connections existing between elements may be traced back, resulting in insights regarding causative and perpetuating factors of a presentation. Depending on individual factors, these may constitute health disturbing triggers best removed or the existence of modifiable treatment targets that when altered will potentially more profoundly resolve the presenting condition as compared with that afforded by specific linear treatment targeting an overt population generalisable symptomotology. Additionally, the presence of multiple linked elements may alert the clinician to the existence of potentially problematic sub-clinical disease processes in action. A systems approach creates the possibility for treatment to be tailored to the individual, causative rather than reactive, and more profound by encompassing the entire human organism in its scope. While this study has focused on incorporating a systems science perspective within primary health care, it may be applicable to any setting that is engaged with managing and supporting human health.

While linear systems in nature are rare [13], the reductionist and mechanistic defining of biomedicine has simultaneously simplified the healthcare task and enabled impressive medical advances [2]. However, continuing to ignore the complex nature of the human organism limits ongoing capacity to resolve contemporary health priorities such as systemic, chronic disease which tends to be multifactorial and complex [4, 7]. In a reductionistic and linear analysis of a case presentation, a list of symptoms may be elicited which correspond with a disease label, prompting selection of a specific treatment to counter this symptom set or condition [4, 5]. Western scientific thinking promotes attention on body components rather than their interrelationships, therapeutic efforts are directed at disease rather than the individual, and the meaning of illness is derived from pathology common to populations [40]. While syndromic patterning offers the advantage of reducing clinical complexity, it also limits the extent to which disease assessment and treatment can be individualised, and reduces identification of risk susceptibility and sub-clinical disease manifestations [7, 41, 42]. A complexity perspective changes the clinical encounter from identification of a disease label and its corresponding treatment [2] to a broader and more contextual understanding of the multiple systems-based connections in play. The potential afforded is to shift the focus of the healthcare model from pathogenesis to salutogenesis; from a model that is reactive to syndromic patterns and disease labels to one that is focused on the (re)creation of health for the individual patient [43, 44].

Health management is a sophisticated and multifaceted task given the complexity of the human organism, the influence of environmental factors on health, and the prevalence of chronic illness, comorbidities, and polypharmacy. Clinicians are sifting and evaluating vast amounts of intricate information, and the efficacy and safety of healthcare is largely dependent upon this process. Evidence based practice (EBP) is founded on patient values, best available evidence, and clinician expertise [45, 46]. Ooi et al. [45] outline potential strengths of EBP including consistency of care, resource conservation, knowledge gap identification, and a patient-focused approach. They also identify limitations of EBP including reduced treatment options, less focus on clinician creativity and professional judgement, overemphasis on evidence from randomised controlled trials and systematic reviews, and inability to respond adequately to unique patients presenting with complex needs [45]. It is possible that inclusion of a complexity perspective may support clinical creativity and judgement, increase the quality of available research, and more closely align clinical understanding with the complex nature of the human organism, while strengthening the positive aspects of EBP. A mapping and analysis of the clinical reasoning process provides potential insights into the cognitive processes underpinning clinicians’ decision-making and augments clinician expertise, awareness and reasoning.

### Implications for research

In addition to supporting clinical processes, a network mapping and analysis approach may enable clinical reasoning and practices to be made more explicit for the benefit of the research community. Links made by practitioners between different elements of a patient presentation may be made overt and therefore more amenable to analysis and investigation, increasing scientific research opportunities within the field. Additionally, this facilitates an exploration of the alignment between clinical reasoning and current research, with gaps identified and investigated, and potentially exposes clinician blindspots and biases. Further research based on clinical network mappings may also supplement knowledge of individual conditions, enable exploration of the systematic patterns of chronic conditions, and increase understanding of the symptoms and causative factors common across conditions. Causative chains may also be defined, leading to knowledge advancements in disease prevention and health promotion. While contemporary biomedical research is founded on the concept of identifying a specific treatment for a specific symptom of disease [5] and randomised controlled trials are prioritised [45], a complexity informed approach offers a method fit for the purpose of exploring and understanding health in the context of the whole person. While this study has focused on the feasibility of using complexity tools and strategies to investigate case conceptualisation processes, future research directions potentially include interpreting the results from the perspective of how they may be utilised to support the development of clinicians’ capacity to manage chronic and complex illness.

### Limitations

This paper is limited in that the mind maps created by the participants were constrained by the information contained within the paper case study. While the case study was detailed, it did not replicate the extent of information that typically would be gathered within an initial naturopathic consultation. Also, the combined mapping was generated using mind maps provided by seven naturopaths. While this provides preliminary insight into how complexity tools and strategies might be utilised within the clinical setting, it does not provide a definitive understanding of this process. At best this data could be considered indicative rather than representative, although it was deemed sufficient for the purpose of an introductory exploration of the alignment between clinical health management and complexity science. While naturopathy was chosen for this initial exploratory study due to its stated philosophical and practice framework aligning with complexity science, it is unknown how useful this methodology may be for other health professions without first testing it within these populations, suggesting a possible future avenue for research of this nature.

## Conclusion

While this is an preliminary demonstration of the use complexity tools to investigate the clinical management of a patient case, it is possible that with further use and development new insights will emerge that will improve healthcare practices and treatment outcomes. Medicine has gained considerable ground in viewing the human organism as being composed of discrete components, distinct functional systems, and organised in linear causal chains. The next stage of development may be to explore, understand and relate to the human organism as a complex system, embedded within larger complex systems, and with multiple smaller systems nested within. This complexity perspective does not need to occur at the expense of reductionist knowledge and understanding, but rather allows the potential for the integration of both. The value of network mapping and analysis is to deepen the understanding of the network being explored; a complexity perspective of health and the human organism offers potential benefits for both clinical practitioners and researchers. The findings of this study highlight the clinical reasoning underpinning the management of a case, and potentially offers insights into clinician knowledge and expertise that could tested against current research as well as used to inform future research.

## Supplementary Information

**Additional file 1.**

**Additional file 2.**

**Additional file 3.**

**Additional file 4.**

## Data Availability

All data generated or analysed during this study are included in this published article (and its supplementary information files).

## Abbreviations

CAS: Complex adaptive systems
EBP: Evidence based practice
EDA: Exploratory data analysis
WHO: World health organisation
