# Supplementary material for: Primary health care case management through the lens of complexity: an exploratory study of naturopathic practice using complexity science principles
Source: BMC Complement Med Ther. 2022 Apr 15;22:107. doi: 10.1186/s12906-022-03585-2 (PMC9011958; doi:10.1186/s12906-022-03585-2)
Supplement: Supplementary file 1 — Additional file 1. [file 12906_2022_3585_MOESM1_ESM.docx]

## Supplementary file 1: case study

Male, 35 years old.

Presenting issue: would like to lose weight (GP’s recommendation)

BP: 135/85

Height: 187cm, Weight: 122kg, BMI: 34.9, Waist measurement: 112cm

GP has prescribed an SSRI for flat mood / mild depression, which client has been on for 5 weeks. As yet hasn’t noticed any improvement. GP has suggested counselling but client feels he wouldn’t know what to say.

**Case notes:**

- Unenthusiastic about job (accountancy). Same company and role for over 10 years. Feels is being passed over for promotion.
- Shares a house with two other people. They like each other but don’t have a lot in common and tend to keep to themselves.
- Walks 15 minutes to and from train station Monday to Friday.
- Plays indoor cricket once a week (social game) and then a few beers afterwards. Team mates are “a good bunch of blokes but we don’t catch up at other times”.
- Often goes fishing on the weekend either with a mate or on his own.
- Likes a night at home watching a movie (has tv in bedroom) or video gaming.
- Last partner was 7 years ago. Would like a girlfriend but doesn’t seem to meet any suitable women. Tried an online dating site but after a couple of awkward and unsuccessful dates has given up on that approach.
- Doesn’t enjoy parties and feels uneasy / anxious in groups of people he doesn’t know well.
- No suicidal ideation but does wonder “what’s the point?”. Doesn’t feel that life is very enjoyable but more “just going through the motions”.
- Knows he should be doing things to improve his life but lacks ideas and motivation.
- Bristol Stool Chart type 5 to 6. Bowel movement (BM) 1 to 2 times most days without issue. Stools smell bad (house mates make comments).
- Feels that he has more wind than most people.
- Occasional cramping pain in gut (1 to 2 times a week), that is relieved by passing wind or BM.
- Bloating after meals, relieved by burping or passing wind.
- Heart burn if he has spirits or more than a few beers, or eats too fast.
- Sweats a lot which causes him embarrassment at work.
- Concerned that he has bad breath, often has a bad taste in mouth.
- Sleeps without issue. In bed by midnight, gets up at 7am.
- Headache 4 to 5 times a month which he relieves with neurofen plus. Thinks it might be due to sitting at desk on the computer.

**Diet:**

Client’s standard diet which is consistent and unchanging over past 10 plus years.

Breakfast: muffin or scone, plus 2 to 3 café lattes (cow’s milk plus 2 sugars) across the morning.

AM Snack: “a few” sweet biscuits.

Lunch: sushi, salad sandwich, schnitzel sandwich or burger, plus can of coke.

PM Snack: “a few” sweet biscuits or a chocolate bar. Occasionally another can of coke.

Dinner: either take away (sweet and sour pork with rice, green chicken curry with rice, meat lovers pizza or lamb souvlaki) or a meal at the pub (burger or chicken parmigiana). Occasional bbq with house mates.

2 to 3 beers in the evening, maybe more on the weekend.

Supper: bag of potatoe chips or savoury shapes when watching a movie / gaming.

Water: Up to 3 glasses per day, occasionally none.

**Instructions:**

Create a mind map according to the process that you usually use. There are no right or wrong answers. Your mind map can be created using mind mapping software or hand drawn. The aim is to provide a sense of your process and the style of mind mapping that you use.
