# Supplementary material for: Primary health care case management through the lens of complexity: an exploratory study of naturopathic practice using complexity science principles
Source: BMC Complement Med Ther. 2022 Apr 15;22:107. doi: 10.1186/s12906-022-03585-2 (PMC9011958; doi:10.1186/s12906-022-03585-2)
Supplement: Supplementary file 2 — Additional file 2. [file 12906_2022_3585_MOESM2_ESM.docx]

Supplementary file 2: Degree for all elements within the network


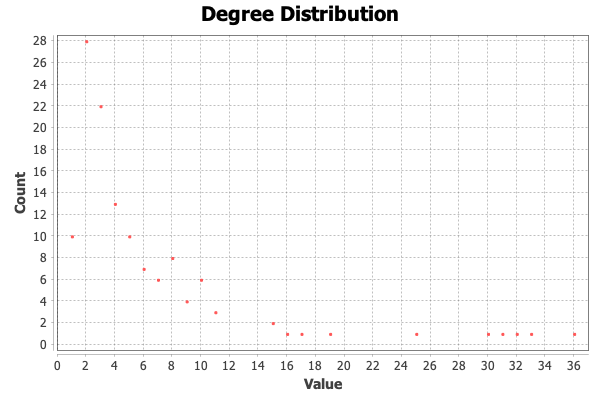


Value = number of links each element has to other elements in the network (degree)

Count = number of elements with specified number of links

Elements with high degree values:

| Element label | Degree |
| --- | --- |
| Dysbiosis | 36 |
| Diet | 33 |
| Flat mood / depression | 32 |
| Obesity | 31 |
| Systemic inflammation | 30 |
| Liver | 25 |
