# Supplementary material for: Primary health care case management through the lens of complexity: an exploratory study of naturopathic practice using complexity science principles
Source: BMC Complement Med Ther. 2022 Apr 15;22:107. doi: 10.1186/s12906-022-03585-2 (PMC9011958; doi:10.1186/s12906-022-03585-2)
Supplement: Supplementary file 3 — Additional file 3. [file 12906_2022_3585_MOESM3_ESM.docx]

Supplementary file 3: Betweenness Centrality Distribution


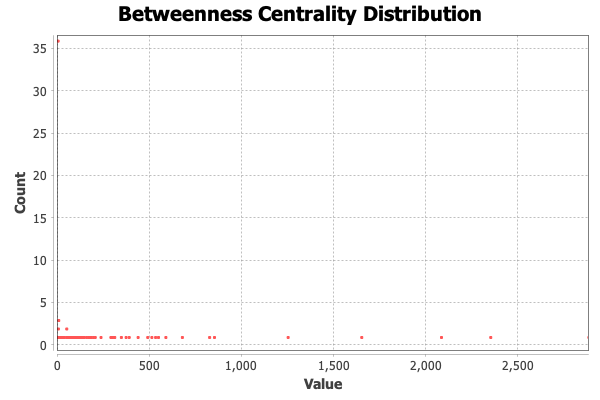


Elements with highest betweenness centrality values:

| Element label | Value |
| --- | --- |
| Flat mood / depression | 2880 |
| Obesity | 2348 |
| Dysbiosis | 2080 |
| Systemic inflammation | 1648 |
| Liver | 1248 |
