# Supplementary figures and images for: Primary health care case management through the lens of complexity: an exploratory study of naturopathic practice using complexity science principles
Source: BMC Complement Med Ther. 2022 Apr 15;22:107. doi: 10.1186/s12906-022-03585-2 (PMC9011958; doi:10.1186/s12906-022-03585-2)

Supplementary file 4: Eigenvector Centrality Distribution


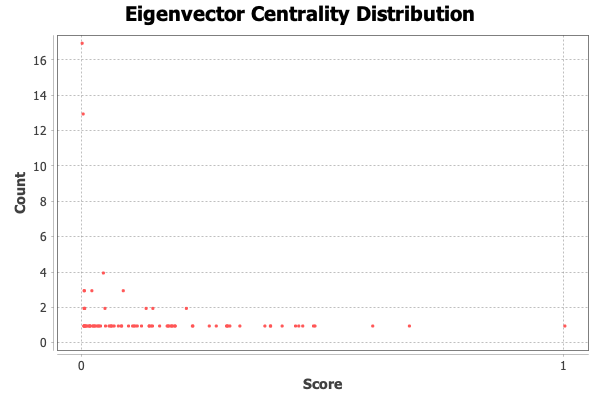

Supplement: Supplementary file 4 — Additional file 4. [file 12906_2022_3585_MOESM4_ESM.docx]
